# Adult Height, 22q11.2 Deletion Extent, and Short Stature in 22q11.2 Deletion Syndrome

**DOI:** 10.3390/genes13112038

**Published:** 2022-11-05

**Authors:** Tracy Heung, Brigid Conroy, Sarah Malecki, Joanne Ha, Erik Boot, Maria Corral, Anne S. Bassett

**Affiliations:** 1 Clinical Genetics Research Program, Centre for Addiction and Mental Health, Toronto, ON M5S 2S1, Canada; 2 The Dalglish Family 22q Clinic, University Health Network, Toronto, ON M5G 2C4, Canada; 3 Undergraduate Medical Education, Faculty of Medicine, University of Toronto, Toronto, ON M5S 1A4, Canada; 4 Department of Medicine, University of Toronto, Toronto, ON M5S 1A4, Canada; 5 Advisium, ’s Heeren Loo, 3818 LA Amersfoort, The Netherlands; 6 Department of Psychiatry, University of Toronto, Toronto, ON M5S 1A4, Canada; 7 Toronto General Hospital Research Institute, Toronto, ON M5G 2C4, Canada; 8 Campbell Family Mental Health Research Institute, Toronto, ON M5G 2C1, Canada

**Keywords:** copy number variation, DiGeorge syndrome, velocardiofacial syndrome, adult phenotype, quantitative trait

## Abstract

The 22q11.2 deletion syndrome (22q11.2DS) manifests as a wide range of medical conditions across a number of systems. Pediatric growth deficiency with some catch-up growth is reported, but there are few studies of final adult height. We aimed to investigate how final adult height in 22q11.2DS compared with general population norms, and to examine predictors of short stature in in a cohort of 397 adults with 22q11.2DS (aged 17.6–76.3 years) with confirmed typical 22q11.2 microdeletion (overlapping the LCR22A to LCR22B region). We defined short stature as <3rd percentile using population norms. For the subset (*n* = 314, 79.1%) with 22q11.2 deletion extent, we used a binomial logistic regression model to predict short stature in 22q11.2DS, accounting for effects of sex, age, ancestry, major congenital heart disease (CHD), moderate-to-severe intellectual disability (ID), and 22q11.2 deletion extent. Adult height in 22q11.2DS showed a normal distribution but with a shift to the left, compared with population norms. Those with short stature represented 22.7% of the 22q11.2DS sample, 7.6-fold greater than population expectations (*p* < 0.0001). In the regression model, moderate-to-severe ID, major CHD, and the common LCR22A-LCR22D (A-D) deletion were significant independent risk factors for short stature while accounting for other factors (model *p* = 0.0004). The results suggest that the 22q11.2 microdeletion has a significant effect on final adult height distribution, and on short stature with effects appearing to arise from reduced gene dosage involving both the proximal and distal sub-regions of the A-D region. Future studies involving larger sample sizes with proximal nested 22q11.2 deletions, longitudinal lifetime data, parental heights, and genotype data will be valuable.

## 1. Introduction

The 22q11.2 deletion syndrome (22q11.2DS) is the most common of the clinically relevant microdeletion syndromes, affecting males and females about equally, with estimated live birth prevalence of the 22q11.2 microdeletion approaching 1 in 2000 [1,2,3]. This multi-system condition is associated with a broad range of developmental and later onset features of varying severity, including congenital cardiac and palatal anomalies, intellectual disabilities, hypocalcemia, hypothyroidism, and significant neuropsychiatric conditions, such as schizophrenia and early onset Parkinson’s disease [1,2,3]. The phenotypic manifestations of the syndrome are thought to be related at least in part to the reduced dosage of genes in the 2.5 million base pair 22q11.2 deletion region [1,2]. Pediatric short stature is reported to be a relatively frequent finding in children, and pediatric growth curves are available for those of European ancestry with 22q11.2DS [4]. However, data on final adult height are limited [4,5,6].

There is a general association of pathogenic copy number variation (CNV) with short stature reported in the pediatric genetic literature [7,8,9] and height-lowering effects on the average of CNVs, especially deletions, in adult data [10,11]. However, none of the samples studied included more than a few individuals with a 22q11.2 microdeletion. Additionally, to our knowledge, there are no studies of factors that may relate to short stature in adults with 22q11.2DS.

Therefore, in a large cohort of adults with a 22q11.2 microdeletion [12], we compared adult height to population-based norms, and examined possible predictors of short stature, including the extent of the 22q11.2 deletion. Based on the general literature, we hypothesized that moderate-to-severe intellectual disability (ID) [13,14] and major congenital heart disease (CHD) [15,16] may be significant predictors of short stature.

## 2. Materials and Methods

### 2.1. Participants and Study Design

**Figure 1:**
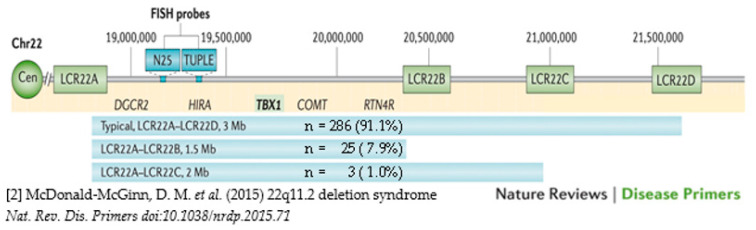
The common types of 22q11.2 deletion extent within the LCR22A-LCR22D region and the frequencies of each for the *n* = 314 adults used in regression analyses.

The cohort studied comprised a well-characterized sample of adults (age > 17 years) with 22q11.2DS (*n* = 397; 192 M, 205 F; median age 32.3, range 17.6–76.3, years) who had data available on adult height. We excluded a single female participant with a history of growth hormone deficiency and severe scoliosis where recorded height was 128 cm, representing an extreme outlier. All individuals had a typical 22q11.2 deletion on clinical genetic testing by fluorescence in situ hybridization (FISH) and a probe (usually TUPLE1), targeting the proximal 22q11.2 deletion region or by genome-wide microarray [1,2]. For those with only FISH data, there are no data on 22q11.2 deletion extent (*n* = 83, 20.9%) but for the majority (*n* = 314, 79.1%) we had data on whether the 22q11.2 deletion was the common 2.5 Mb LCR22A-LCR22D (A-D) deletion, or the rarer proximal nested LCR22A-LCR22B (A-B) or LCR22A-LCR22C (A-C) deletions (Figure 1).

The most recent adult height, measured using a stadiometer, was used for this study. We recorded demographic variables (sex, age at height measurement, ethnicity) and clinical variables, including major CHD (defined as moderate-to-complex structural/anatomic complexity [17]), and moderate-to-severe ID [18,19].

The study was approved by the local research ethics boards of the Centre for Addiction and Mental Health and University Health Network, which are affiliated with the University of Toronto. Informed consent was obtained in writing for participants.

### 2.2. Analyses

We used Canadian WHO growth charts to obtain population-based height norms (https://www.dietitians.ca/Advocacy/Interprofessional-Collaborations-(1)/WHO-Growth-Charts/WHO-Growth-Charts-Set-2, accessed on 15 May 2022), using data for the eldest available (at age 19 years), for males and females. These data provided normal distributions, means, standard deviations (SD), and percentile values for adult height. We used a standard definition of short stature as height < 3rd percentile of population norms, i.e., 2 SDs below the corresponding mean height of a given age, sex, and population group [20].

For descriptive statistics and univariate comparisons, we used parametric and non-parametric analyses as appropriate. We compared the 22q11.2DS results for adult height to the population norms, first using the Shapiro–Wilk test of normality to determine whether the 22q11.2DS heights were normally distributed. We also used the population height norms for males and females to determine the percentage of individuals in the 22q11.2DS cohort who met criteria for short stature.

We used a binomial logistic regression model to investigate the following possible demographic and clinical predictors of short stature in 22q11.2DS: moderate-to-severe ID, major CHD, non-European ethnicity, age (in 10-year increments), male sex, and 22q11.2 deletion extent. For this analysis, we used the subgroup of 314 individuals with data on 22q11.2 deletion extent in order to assess whether the most common A-D deletion had a greater effect than shorter proximal nested deletions. We conducted multicollinearity analyses to identify any potentially correlated variables in the models tested; variance inflation values did not exceed 1.2, suggesting there were no issues with multicollinearity. In post hoc analyses, we assessed possible effects of scoliosis and (treated) hypothyroidism, adding each of these variables, individually, to the original logistic regression model.

All statistical analyses were conducted with SAS 9.4 (SAS Institute, Cary, NC). We defined statistical significance as *p* < 0.05, two-tailed. All analyses, except those using the regression models, used the entire cohort of 397 individuals with 22q11.2DS.

## 3. Results

**Figure 2:**
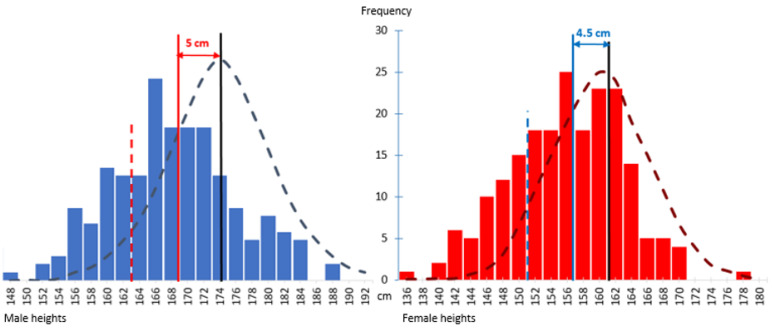
The histogram on the left (blue bars) shows a normal distribution for adult heights of 192 male 22q11.2DS participants, with a mean (red vertical line) of 169.0 ± 7.8 cm, and on the right the histogram (red bars) similarly shows a normal distribution for adult heights of 205 female 22q11.2DS participants, with mean (blue vertical line) 156.7 ± 7.1 cm. The superimposed male (mean 174.1 ± 7.3 cm) and female (mean 161.2 ± 6.2 cm) Canadian population adult height normal curves for males and females are shown, respectively, by long dashed dark blue and dark red lines. Both historgrams show a leftward shift of approximately 5.0 and 4.5 cm for males and females with 22q11.2DS, respectively. Bars to the left of the dashed red/blue vertical lines indicate those meeting criteria for short stature for males and females, respectively.

The mean adult height of the 397 individuals with 22q11.2DS was 169.0 ± 7.8 cm for the 192 males and 156.7 ± 7.1 cm for the 205 females. Mean height was significantly less than for adult population norms for both sexes (t-score 4.86 for males, 4.82 for females, *p* < 0.0001 for both). Graphical portrayals of the distributions of adult heights for 22q11.2DS by sex show normal distributions (Shapiro–Wilk test of normality W = 0.99, *p* = 0.5092 for males, W = 0.99, *p* = 0.4325 for females), with average leftward shifts of approximately 5.0 ± 0.7 and 4.5 ± 0.6 cm for males and females, compared to population-based norms (Figure 2), respectively.

There were 90 (22.7% of 397) individuals with 22q11.2DS who met the criteria for short stature; these comprised 43 (47.8%) males and 47 (52.2%) females, indicating no sex differences (*p* = 0.8995). The overall proportion of the adult 22q11.2DS sample with short stature was 7.6-fold greater than population expectations (*p* < 0.0001).

Table 1 summarizes results of univariate analyses of demographic and clinical variables, comparing those with and without short stature for the subgroup of 314 individuals with data on 22q11.2 deletion extent. As hypothesized, individuals with moderate-to-severe ID and to a lesser extent those with major CHD had a statistically significantly higher proportion with short stature. Results were also significant for those with the proximal nested 22q11.2 deletion, compared with those who had a full-length deletion but in the opposite direction of effect. Results were non-significant for sex and age and were at the non-significant trend level for those of non-European ethnicity. There was no significant difference in height between the 314 with molecular results (and thus known 22q11.2 deletion extent data), compared with the 83 for whom only FISH results were available (mean height 162.8 and 161.6 cm, respectively, z = 0.48, *p* = 0.6310).

**Figure 3:**
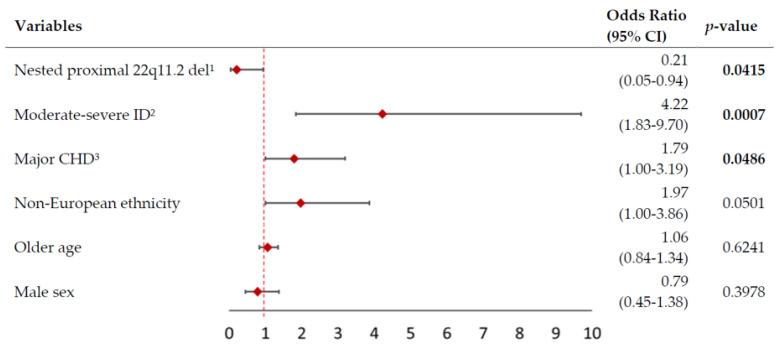
Forest plot of logistic regression model for short stature in 314 adults with 22q11.2DS. The figure shows odds ratios (OR, red diamonds) and 95% confidence intervals (CI, horizontal lines) for six possible predictors of short stature in 314 adults with a 22q11.2 microdeletion and known deletion extent. The overall logistic regression model was significant (likelihood ratio χ^2^ = 24.5, df = 6, *p* = 0.0004). Significant values are shown in bold font. ^1^ Nested proximal 22q11.2 deletions (A-B or A-C; see Methods). ^2^ ID = intellectual disability (see Methods). ^3^ CHD = congenital heart disease (see Methods).

The logistic regression model was statistically significant (likelihood ratio χ^2^ = 24.5, df = 6, *p* = 0.0004). As hypothesized, moderate-to-severe ID and major CHD were significant independent predictors of short stature (Figure 3). The model also indicated that those with a proximal nested 22q11.2 deletion, rather than the full length 22q11.2 deletion, were at a significantly lower risk of having adult short stature, even when accounting for the other predictors (Figure 3). Regression results remained significant for the proximal nested 22q11.2 deletion and ID variables when either hypothyroidism or scoliosis were added to the model (likelihood ratio χ^2^ = 24.3, df = 6, *p* = 0.0006; likelihood ratio χ^2^ = 25.3, df = 6, *p* = 0.0003 respectively); however, results for major CHD no longer reached significance in either of these post hoc models (*p* = 0.0601 and *p* = 0.0626 respectively).

There was no evidence that these results could be mainly attributed to major pediatric growth conditions. One male participant with a history of growth hormone deficiency as a child had a final adult height at about the 10th percentile per Canadian male norms; another who received human growth hormone therapy (at age 15–17 years) for delayed puberty was in the short stature subgroup. For eight individuals with a history of failure to thrive as infants, five (one male, four female) were in the short stature subgroup and three had adult heights that were either in the <10th percentile (*n* = 2) or >10th percentile (*n* = 1).

## 4. Discussion

The results of this study support a significant effect of the 22q11.2 microdeletion on adult height. Stature in adults with 22q11.2DS appears to have an overall normal distribution, with a significant shift to the left (lower adult height), compared to population norms, and including a substantial subgroup with short stature defined conservatively as <3rd percentile. There were several other novel findings that begin to help us understand possible factors associated with short stature in 22q11.2DS. These include moderate-to-severe ID and what appear to be lesser though significant independent associations with major CHD, and with the length of the 22q11.2 microdeletion itself.

Approximately 22.7% of adults with 22q11.2DS met criteria for short stature. This was a similar proportion to that previously reported in an earlier study involving a much smaller adult sample [21]. The height results are comparable to, but extend, previous primarily pediatric data available on stature in 22q11.2DS [4]. Collectively, the data reflect that while a substantial minority have short stature, many individuals with a 22q11.2 microdeletion have height well in the normal range, and some are taller than average.

The association of short stature with the rare subgroup of 22q11.2DS who have moderate-to-severe ID is consistent with long term evidence for an association between growth and neurocognitive abnormalities in the general population [13,14,22,23]. There was also some evidence of association of short stature with severe CHD. Reassuringly, there were no significant effects of age or sex on the short stature results. However, even though not reaching significance, the results suggest that ethnicity will be an important consideration in future studies, given the known effects on height [24,25].

The findings in regard to significant effects of length of the 22q11.2 microdeletion on the likelihood of short stature provide intriguing molecular implications. This is one of the few examples so far identified of differing effects related to 22q11.2 deletion extent [26]. The full length ~2.5 Mb 22q11.2 A-D deletion conveyed significantly greater risk of adult short stature than the shorter, proximal nested 22q11.2 deletions (A-B or A-C) that affected a minority (8.9%) of the sample. This suggests the possibility that gene dosage effects from both the proximal nested A-B and distal nested B-D sub-regions may contribute to the low end of final height in 22q11.2DS.

These results for adult height in 22q11.2 microdeletion may now be placed in the context of previous studies of rare CNVs. In general, these have shown an association between short stature and rare CNVs, especially for deletions [7]. There are also several reports of general effects of CNVs on height in multi-cohort studies [10], often combining effects of all rare CNVs rather than examining individual CNVs. This is due to power considerations, especially for high impact CNVs that are disproportionately rare in these samples in part due to ascertainment issues [11]. Notably, none of these studies included more than 11 individuals with a 22q11.2 microdeletion [7]. For example, a study using UK Biobank data reported that the 10 individuals with a 22q11.2 microdeletion (of unknown sex or age) in the sample had significantly shorter stature (on average by 0.58 Z) than 380,630 other participants [11].

***Advantages and limitations***: We present results on what is, to our knowledge, the largest dataset available of adult height in 22q11.2DS. The sample provided sufficient power to delineate several novel findings and to confirm the strong effects of the 22q11.2 microdeletion on adult height and short stature. We excluded data on only one individual representing an extreme outlier, and included individuals with treated hypothyroidism and scoliosis, with neither factor appearing to significantly affect the results. In 22q11.2DS, few children have a formal diagnosis of growth deficiency [2], consistent with our findings. Larger numbers of such individuals would be necessary to evaluate this as a factor in adult short stature. Additionally, there were insufficient numbers of individuals of non-European ancestry to see significant results for this important variable.

***Implications and future directions***: Results from the current study further strengthen a recent American College of Medical Genetics (ACMG) revision to 2009 guidelines on genetic evaluation of short stature, that advises genome-wide microarray as a first line investigation [27]. Further studies of adult stature in 22q11.2DS are needed, especially those that include larger samples of the rare proximal nested 22q11.2 deletions, of non-European ancestries, and with intra-individual longitudinal data to delineate growth trajectories. Possible factors to be considered may include hormonal effects, however, there is no evidence that puberty is a significant factor in 22q11.2DS [4]. As for IQ in 22q11.2DS [28], assessing height in the context of parental heights will be important; this has been shown to contribute to predicting adult height in the general population and to assist with estimating genetic contributions [29].

Next steps should also include investigation of the role of genetic variants within the 22q11.2 microdeletion region and genome-wide, including common variants and polygenic risk score for height, and rare variants of all types [29,30,31]. This will be important as there may be effects of reduced gene dosage in the 22q11.2 deletion region that could affect growth from conception onward [3], and this may add to or interact with genome-wide variants for height. There is evidence for such a mechanism in 22q11.2DS for other phenotypes [32]. The 22q11.2 deletion region was not highlighted in a recent study identifying clusters of common variants across the genome that affect height [31]. However, conclusions regarding regional enrichment of biologically relevant genes may be consistent with our findings for 22q11.2DS. Genes located in the 22q11.2 deletion region, such as *DGCR8* involved in microRNA processing, the several related to mitochondrial functions, and others, may affect growth [33]. The results of the current study foreshadow a further role of the 22q11.2 microdeletion in understanding the complex pathways underlying human growth and stature.

## Figures and Tables

**Table 1:** Clinical and demographic factors for adults with 22q11.2 deletion syndrome for the subgroup of *n* = 314 with known 22q11.2 deletion length.

Variables	Subgroup with Known Deletion Length (*n* = 314)		Short Stature		Statistics
			Yes (*n* = 71)	No (*n* = 243)	
Categorical	*n*	%	*n* (%)	*n* (%)	*p*-value ^2^
Moderate–severe intellectual disability	28	8.9	14 (19.7)	14 (05.8)	0.0003
Major congenital cardiac disease	104	33.1	31 (43.7)	73 (30.0)	0.0319
Nested proximal 22q11.2 deletion ^1^	28	8.9	2 (02.8)	26 (10.7)	0.0403
Non-European ancestry	67	21.3	20 (28.2)	47 (19.3)	0.1102
Male sex	155	49.4	33 (46.5)	122 (50.2)	0.5806
**Continuous**			**Median (range)**		
Median age (range) at height (years)	314	100	34.0 (19.0–67.3)	35.0 (18.0–76.3)	0.5537

^1^ A,B and A–C (vs. full A–D) 22q11.2 deletions. ^2^ *p* values are from χ^2^ analysis (df = 1) for categorical variables, and Wilcoxon for age.

## Data Availability

The data are not publicly available due to ethical restrictions and privacy concerns. Any data requests can be directed to the corresponding author.
